# UiO-66 nanoparticles combat influenza A virus in mice by activating the RIG-I-like receptor signaling pathway

**DOI:** 10.1186/s12951-024-02358-y

**Published:** 2024-03-09

**Authors:** Ruijing Su, Xinsen Li, Jin Xiao, Jiawei Xu, Jijing Tian, Tianlong Liu, Yanxin Hu

**Affiliations:** 1 National Key Laboratory of Veterinary Public Health and Safety, Key Laboratory of Animal Epidemiology of Ministry of Agriculture and Rural Affairs, College of Veterinary Medicine, China Agricultural University, No. 2 Yuanmingyuan West Road, Beijing, 100193 China; 2 Key Laboratory of Veterinary Bioproduction and Chemical Medicine of the Ministry of Agriculture, Zhongmu Institutes of China Animal Husbandry Industry Co., Ltd, Beijing, People’s Republic of China

**Keywords:** Influenza A virus, UiO-66 nanoparticle, Cytokine storm, RIG-I-like receptor signaling pathway, Antiviral nanomedicine

## Abstract

The Influenza A virus (IAV) is a zoonotic pathogen that infects humans and various animal species. Infection with IAV can cause fever, anorexia, and dyspnea and is often accompanied by pneumonia characterized by an excessive release of cytokines (*i.e.*, cytokine storm). Nanodrug delivery systems and nanoparticles are a novel approach to address IAV infections. Herein, UiO-66 nanoparticles (NPs) are synthesized using a high-temperature melting reaction. The in vitro and in vivo optimal concentrations of UiO-66 NPs for antiviral activity are 200 μg mL^−1^ and 60 mg kg^−1^, respectively. Transcriptome analysis revealed that UiO-66 NPs can activate the RIG-I-like receptor signaling pathway, thereby enhancing the downstream type I interferon antiviral effect. These NPs suppress inflammation-related pathways, including the FOXO, HIF, and AMPK signaling pathways. The inhibitory effect of UiO-66 NPs on the adsorption and entry of IAV into A549 cells is significant. This study presents novel findings that demonstrate the effective inhibition of IAV adsorption and entry into cells via UiO-66 NPs and highlights their ability to activate the cellular RIG-I-like receptor signaling pathway, thereby exerting an anti-IAV effect in vitro or in mice. These results provide valuable insights into the mechanism of action of UiO-66 NPs against IAV and substantial data for advancing innovative antiviral nanomedicine.

**Graphical Abstract:**

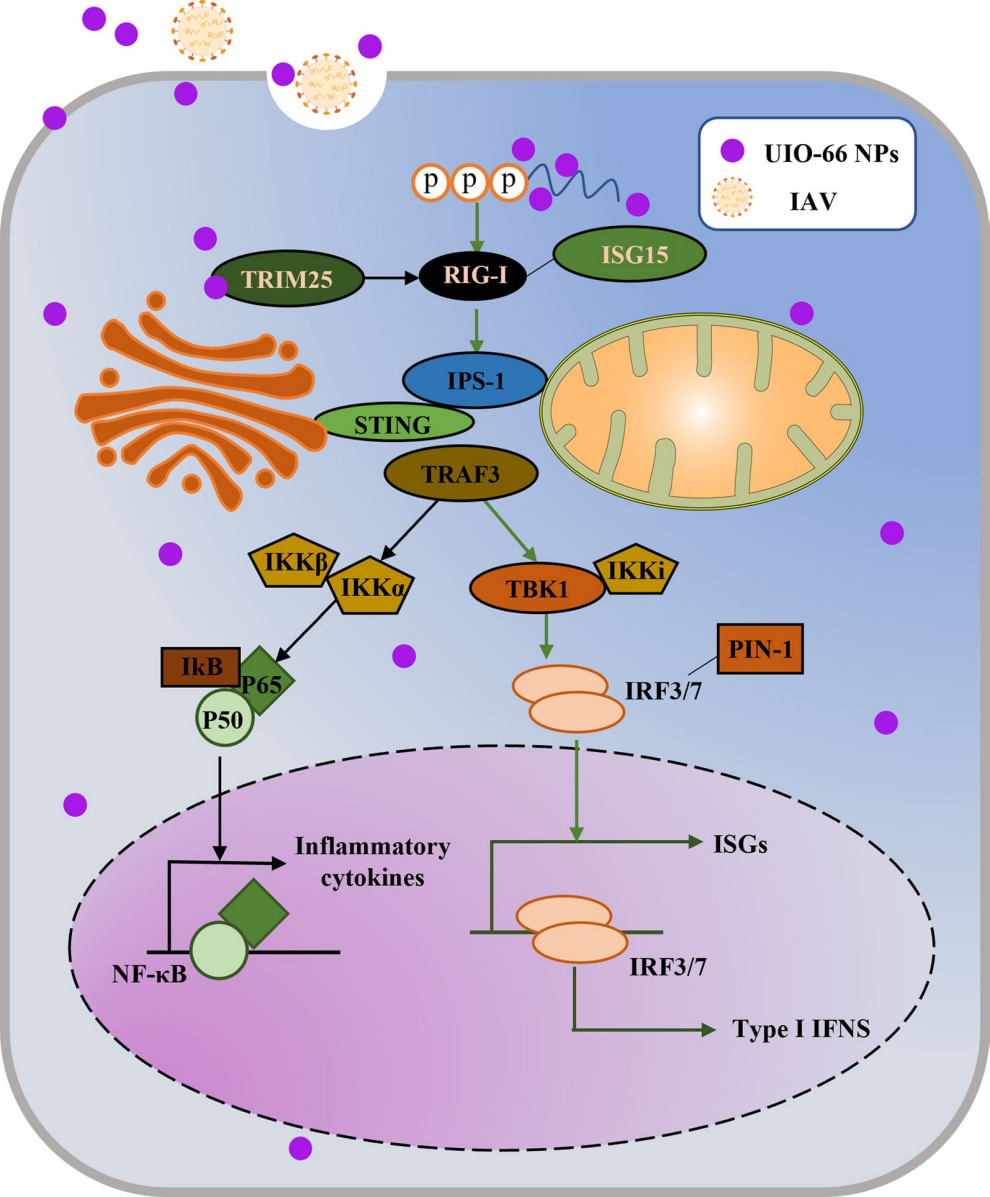

**Supplementary Information:**

The online version contains supplementary material available at 10.1186/s12951-024-02358-y.

## Introduction

In recent decades, nanoparticle technology has become popular in science, exhibiting a wide range of applications [1, 2]. The abilities of nanomaterials have initiated several opportunities for industrial and scientific research because nanotechnology enables the manipulation of performance at an extremely small scale [3]. Nano-technology has been widely used in agriculture, gene delivery, imaging, artificial implantation, and other areas [4–10]. Nanomaterial in medicine is based on its small size, which enables it to enter live cells, particularly human cells. In addition, nanomaterials prevent the degeneration of parcels or anti-infection agents because of their shielding characteristics [11]. Nanoparticle play an important role in cancer treatment and in the manufacturing of potential drugs against bacterial, fungal, and viral infections [12]. Metal–organic framework (MOF) materials are low-density crystalline porous materials comprising organic ligand-linked metal ions that can be used in pharmaceutical and biomedical applications, including antibacterial, drug delivery, and anti-cancer.

IAV causes acute respiratory inflammatory disease in humans, birds, and other mammals and is a major threat to public health. IAVs (i.e., HIN1, H2N2, and H3N2) have caused four influenza pandemics since 1918 with an unprecedented number of deaths [13]. Throughout the twentieth century, the global annual mortality rate due to influenza virus infections ranged from 250,000 to 500,000. Wild birds serve as reservoirs for the IAV, and avian strains can adapt to human hosts and acquire human-to-human transmissibility via mutations [14]. Although some antiviral drugs such as amantadine, oseltamivir, and zanamivir are currently effective in the treatment of influenza infection, they have also increased the occurrence of drug-resistant strains (H1N1, 2008–2010 [15]; H7N9, 2013 [16]; N2 E119V and I222L mutants, 2016 [17]), greatly affecting its clinical application [18]. Influenza viruses undergo frequent mutations owing to their segmented RNA genomes, rendering it impossible to timely produce sufficiently effective vaccines to prevent possible epidemics of drug-resistant virus stains [19]. Due to the rapid diffusion of the virus and its ability to cause gene mutations, viral infection is a potential threat to humans. Therefore, seeking new anti-influenza medications is important.

UiO-66 NPs, a subclass of MOFs with a 12-hedral structure, are synthesized using zirconium tetrachloride and terephthalic acid. They offer various advantages such as good dispersion, large specific surface area, porosity, and convenient synthesis [20]. UiO-66 NPs have been widely used in wastewater adsorption, drug delivery, catalytic reactions, and other applications [20–23]. Compared with several nanomaterials, such as ZnO, copper-oxide, ZIF-8, SnO_2_@ZIF-8, UiO-66 NPs have higher biocompatibility and biodegradability, lower cytotoxicity to living cells [24–28], and strong adsorption capacity to proteins [29]. It has been found that the Zirconium-based nanomaterials could alleviate excessive inflammation caused by H5N1 influenza A virus infection [30]. However, no study has reported the use of UiO-66 NPs as antiviral materials.

In the present study, UiO-66 NPs were synthesized and evaluated for their antiviral activity in vivo. We examined the effect of UiO-66 NPs on the viral replication cycle to elucidate how UiO-66 NPs exert their antiviral effects. In addition, a transcriptomic analysis was performed to elucidate the underlying molecular mechanism of UiO-66 NPs against IAV. Finally, we preliminarily explored the possibility of clinical application of Uio66 NPs in vivo using a mouse infection model, in which the nasal administration of UIO-66 NPs could effectively increase the survival, reduce the viral titer and lung injury of H1N1 infected mice. This study aimed to provide a theoretical basis for the development of new antiviral drugs using UiO-66 NPs.

## Experimental section

### Materials

Zirconium tetrachloride (ZrCl_4_) was obtained from Tianjin Chemical Reagent 3 Plant Co., Ltd. (Tianjin, China). Terephthalic acid (TPA) was supplied by Beijing J&K Chemicals Co., Ltd. (Beijing, China). Low melting point agarose was purchased from Invitrogen (Waltham, MA, USA). Zoletil^®^ (Virbac, Carros, France) was supplied by Animal Hospital of China Agricultural University (Beijing, China). Sambucus nigra lectin (SNA, Stained SAα2, 6Gal receptor) and maackia amurensis lectin I (MAL-I, Stained SAα2, 3Gal receptor) were obtained from Vector Laboratories. The mouse-adapted IAV H1N1 (A/WSN/33) was provided by Dr. George F. Gao of the Institute of Microbiology, CAS, China.

### Animals

The 6-week-old female BALB/c mice (body weight: 18–20 g) were purchased from Beijing Vital River Laboratory Animal Technology Co., Ltd. (Beijing, China). They were raised in an environment free of pathogens and with controlled humidity and temperature. All animal experiments were approved by the Institutional Animal Care and Use Committee of the China Agricultural University.

### Synthesis and characterization of UiO-66 NPs

In this study, UiO-66 NPs were prepared using high-temperature melting. Briefly, N, N-dimethylformamide was mixed with glacial acetic acid. TPA (292 mg) and ZrCl_4_ (378 mg) were added to the mixed liquid and dispersed using sonication. The mixture was stirred for 10 h at 120 ℃. After centrifugation (10,000 rpm min^−1^) for 10 min, methanol was added for ultrasonic cleaning three times. After the third sonication, the solution was collected as UiO-66 NPs. The size and morphology of UiO-66 NPs were examined using TEM (HT7700, Hitachi, Japan). The hydrodynamic particle size distribution and zeta potential of the NPs were determined using a Zetasizer (ZS90; Malvern Panalytical, Malvern, UK). The functional groups of UiO-66 NPs were characterized using a Fourier-transform infrared spectrometer (Excalibur HE 3100, Varian, USA). The global distributions of Zr, O, and C were analyzed using a high-resolution transmission electron microscope (JEM-2100, Hitachi, Japan).

### Cell culture

RAW264.7 mouse macrophages, MDCK canine kidney cell, and A549 human non-small cell lung cancer cells were seeded in DMEM supplemented with FBS (10%), penicillin (100 U mL^−1^) and streptomycin (100 mg mL^−1^), then placed in a 37 ℃, 5% CO_2_ sterile incubator.

### Hemolysis and MTT tests

Fresh New Zealand rabbit blood was collected, centrifuged at 1500 rpm for 10 min, and washed with PBS five times to separate the red blood cells. The isolated red blood cells were then mixed with UiO-66 NPs at final concentrations of 25, 50, 100, 200, and 400 µg mL^−1^. Red blood cells treated with 1% Triton X-100 served as positive controls. After incubation at room temperature for 2 h, the cells were centrifuged at 1500 rpm at 4 ℃ for 5 min, and each treated supernatant (100 µL) was added to a new 96-well plate. The absorbance at 492 nm was measured using a microplate reader (Tecan Trading AG, Mannedorf, Switzerland), and the hemolysis rate was analyzed. The MTT assay kit (Solarbio, M1020) was used to detect the toxic effect of UiO-66 NPs, ZrCl_4_ or TPA in cells at 37 ℃ for 48 h according to the manufacturer’s instructions.

### Infection of cells and mice

For the in *vitro* infection experiments, A549 cells were seeded in 6-well plates at 1 × 10^6^ cells mL^−1^. First, the H1N1 virus (MOI = 1) was inoculated into A549 cells, and the cells were incubated for 1 h at 37 ℃. After washing three times with PBS, DMEM supplemented with 1% bovine serum albumin and UiO-66 NPs, ZrCl_4_ or TPA were added to each well and incubated at 37 ℃ for the certain times.

Mice were randomly divided into IAV (positive control), IAV + UiO-66 (40, 80, 160 mg kg^−1^, intranasal administration, i.n.), and mock (PBS) groups (seven mice per group). The mice were anaesthetized with Zoletil^®^ and infected with the H1N1 virus or PBS intranasally. One day later, the mice were treated with UiO-66 NPs in the corresponding administration mode. Survival, body weight, and activity were monitored daily for at least 14 d. On 3 and 6 DPI, three mice were selected randomly from each group (IAV, IAV + UiO-66 (i.n.), IAV + UiO-66 (intraperitoneal injection, i.p.), and mock group) (six mice per group) and euthanized by intramuscular injection with Zoletil^®^ and cervical dislocation. The lung tissues of each mouse were collected, and the left lung was placed in sterile tubes (− 80 ℃) and the right lung in 4% formalin for further experiments.

### Virus plaque assay

Briefly, A549 cells was infected with virus and incubated with UiO-66 NPs (25, 50, 100, 200, and 400 µg mL^−1^) at 37 ℃ for 24 h. The cell supernatant was collected via centrifugation at 10,000 rpm for 10 min. The diluted supernatant was added and incubated with MDCK cells for 1 h. Subsequently, the inoculum was removed and a low-melting 1% agarose mixture containing TPCK-trypsin (1 μg mL^−1^) (SIGMA 4370285, USA) was added to the MDCK cells. After 72 h, agarose was removed by treatment with 4% formaldehyde for 20 min. Cells were stained with 2% crystal violet for 15 min, and viral plaques were counted. PFU/mL = viral plaque count × dilution ratio.

### Inactivation, adsorption, and invasion assays

The IAV was incubated with UiO-66 NPs at 37 ℃ for 2 h. After precooling at 4 ℃ for 30 min, MDCK cells were infected with the pretreated IAV at 4 ℃ for 1 h, followed by plaque assay. For adsorption assays, A549 cells were precooled at 4 ℃ for 30 min and then infected with H1N1 (MOI = 0.1) for 2 h at 4 ℃ in DMEM (2% FBS) containing FITC-labeled UiO-66 NPs. After discarding the supernatant, the immunofluorescence staining of nuclear proteins was performed by washing the cells three times with precooled PBS. For the invasion assay, A549 cells were infected with H1N1 (MOI = 0.1) for 2 h at 4 ℃ after precooling for 30 min at 4 ℃. The supernatant was discarded and, after washing three times with precooled PBS, incubated in DMEM (2% FBS) containing FITC-labeled UiO-66 NPs for 3 h at 37 ℃. The immunofluorescence staining of nuclear proteins was performed after washing the cells three times with PBS.

### Immunofluorescent staining and lectin histochemistry

A549 cells was infected with virus and incubated with UiO-66 NPs at 37 ℃ for 24 h. Cells were fixed with 4% formaldehyde and permeabilized with 0.1% Triton X-100 (Solarbio, T8200). Cells or tissues were blocked with a blocking buffer (Beyotime, p0220, QuickBlock™) for 1 h, incubated at 4 ℃ for at least 16 h with a suitable primary antibody, rinsed three times with PBST (Solarbio, P1033), and incubated at room temperature with a suitable fluorescent dye coupled with a secondary antibody for 1.5 h. Sialic acid receptors on A549 cells after incubation with Cy3-labeled UIO-66 NPs (37 ℃, 1 h) were incubated directly with FITC-labeled SNA and MAL-I for lectin histochemistry. Nuclei were stained with DAPI (Beyotime, C1005) or hoechst 33,342 (Solarbio, C0031). Images were obtained using a confocal microscope (FV10-ASM; Olympus Microsystems, Tokyo, Japan).

### siRNA transfection

Small interfering RNA (siRNA) against RIG-I mRNA was produced by Gene Pharma (Gene Pharma Co., Ltd., China). siRNA (si-NC) was used as a negative control. The transfection protocol was as follows: the contents of two centrifuge tubes, of which one contained si-NC or si-RIG-I with Opti-MEM and the other contained 5 μL Lipofectamine 2000 with Opti-MEM (Invitrogen), reacted for 5 min. The two tubes were mixed and incubated at room temperature for 20 min. The mixture was then added to an A549 cell culture plate for 8 h at 37 ℃.

### Histology and immunochemistry

Tissue sections were de-paraffinized and stained with hematoxylin and eosin. Criteria for grading lung histopathological changes were as follows [31] : 0 = no microscopic lesions; 1 = extremely mild, characterized by mild desquamation of rare bronchial epithelial cells; 2 = mild, characterized by desquamation of rare bronchial epithelial cells, hyperemia; 3 = moderate, characterized by desquamation of bronchial epithelial cells, hyperemia, loosen and obvious edema of blood vessel walls and slight inflammatory cell infiltration; 4 = severe, which is characterized by hyperemia, hemorrhage, loosen and edema of blood vessel wall, more inflammatory cell infiltration and sloughed alveolar epithelial cells. Other tissue sections were immunohistochemically stained using an anti-influenza virus nucleoprotein (NP) monoclonal antibody (AA5H, Abcam) at a 1:1,000 dilution according to the manufacturer’s instructions (Gene Tech, GK600705, China). H1N1 antigen was measured using the number of NP-positive cells per section. All sections were examined using a light microscope (CX31; Olympus, Tokyo, Japan). Results were confirmed by an experienced and qualified pathologist.

### Western blotting

A549 cells infected with H1N1 virus and incubated with UiO-66 NPs at 37 ℃ for 12 h or 24 h and mouse lung tissue ground with magnetic beads at − 20 ℃ were harvested and lysed in RIPA containing 1 mM PMSF for 15 min on ice. The lysate was centrifuged at 12,000 rpm for 10 min. The supernatant was collected and analyzed using a BCA protein assay kit (CW0014S; CWBIO). Denatured protein samples were separated using SDS gel electrophoresis and electrotransferred to a methanol-activated polyvinylidene difluoride membrane (Beyotime, FFP24). The membranes were then soaked in 5% nonfat milk for 1 h at room temperature and incubated overnight with the appropriate primary antibodies in a shaking incubator at 4 ℃. The next day, the membranes were washed three times (5 min each time) with TBST and incubated for 1.5 h at room temperature with an HRP-labeled secondary antibody diluted in a 1% blocking solution. After washing three times with TBST, proteins were detected using a chemiluminescence gel imaging system (Tanon-5200Multi). The grayscale values of the protein bands were analyzed using the ImageJ software. Relative target protein levels were calculated using β-actin as the internal control.

### Quantitative real-time PCR

Total RNA was extracted from A549 cells infected with H1N1 virus and incubated with UiO-66 NPs ZrCl_4_ or TPA at 37 ℃ for 12 h or 24 h and lung tissues using Trizol, as previously described. Primers for the genes are listed in Additional file 1: Table S1.

### RNA sequencing and analysis

RNA degradation and contamination were monitored on agarose gels. RNA purity was determined using a spectrophotometer, RNA concentration was measured using a Qubit, and RNA integrity was assessed using Agilent 2100. A total amount of 2 μg RNA per sample was used as input material for the RNA sample preparations. Sequencing libraries were generated using the VAHTS mRNA-seq v2 Library PrepKit for Illumina following the manufacturer’s recommendations, and index codes were added to attribute the sequences to each sample. The libraries were sequenced on an Illumina NovaSeq platform to generate 150 bp paired-end reads, according to the manufacturer’s instructions. Raw data (raw reads) in FASTQ format were first processed using primary quality control. In this step, clean data (clean reads) were obtained by removing read pairs that contained N > 3, or the proportion of bases with quality values below five was > 20% at any end or adapter sequence were identified. All downstream analyses were based on clean, high-quality data. Differential expression analysis between the two conditions was performed using the DEGSeq R package (version 1.20.0). DEGs were defined as those for which the adjusted P-value was < 0.05, and the log2 (fold-change) was > 1. GO and KEGG enrichment analyses of the DEG sets were implemented in the GOseq R and KOBAS 3.0 packages, respectively. GO terms with adjusted P-value < 0.05 were considered significantly enriched by DEGs.

### Toxicity evaluation of UiO-66 NPs in vivo

Fifteen BALB/c mice with a body weight of 20–22 g were randomly divided into control, intranasal administration, and intraperitoneal injection groups (UiO-66 NPs), with five animals per group. After 14 d, the weight changes of the mice were recorded, and routine blood, biochemical, and histological analyses were performed to determine the level of Zr^4+^ in each organ and evaluate the toxicity of UiO-66 NPs.

### Statistical analysis

All data are presented as mean ± standard deviation. One-way analysis of variance and Duncan's multiple comparison post-hoc tests were performed using GraphPad Prism software (version 9.0; CA, USA). Statistical significance was set at P < 0.05. All experiments were performed at least three times.

## Results

### Characterization of UiO-66 NPs

**Fig. 1:**
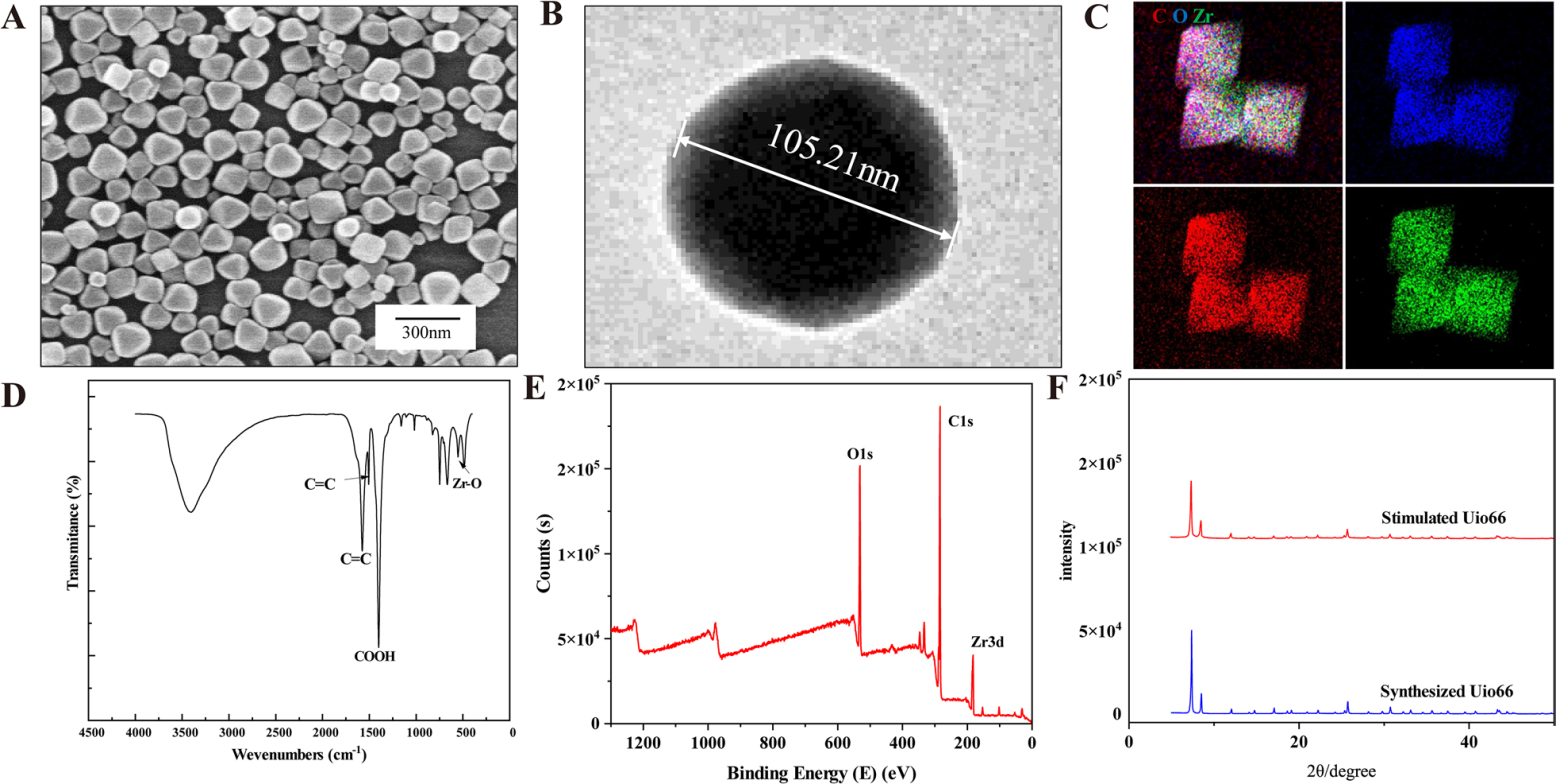
Characterization of UiO-66 NPs. **A** The SEM image of UiO-66 NPs. **B** The TEM image of UiO-66 NPs. **C** The EDS mapping image of UiO-66 NPs. **D** The FTIR spectrum of UiO-66 NPs. **E** The XPS spectroscopy of UiO-66 NPs. **F** The XRD diffraction pattern of UiO-66 NPs

In this study, UiO-66 NPs were synthesized at 120 ℃. Transmission electron microscopy (TEM) showed that UiO-66 NPs had a uniform particle size of approximately 105 nm (Fig. 1A, B). The EDS plot showed that C, O, and Zr were distributed throughout the NPs (Fig. 1C). Fourier-transform infrared spectroscopy (Fig. 1D) revealed crystallization peaks. In addition, the X-ray photoelectron spectroscopy survey scanning spectra clearly showed the presence of C, O, and Zr (Fig. 1E), which is consistent with the EDS elemental map. The hydrodynamic diameter of the UiO-66 NP was 130 nm, as determined by dynamic light scattering (Additional file 1: Fig. S1A), and the zeta potential of the NPs was − 3.06 mV (Additional file 1: Fig. S1B). In addition, the crystallinity of UiO-66 NPs was measured using powder X-ray diffraction, and the peaks of the synthesized UiO-66 NPs were consistent with those of standard UiO-66 NPs (Fig. 1F). These results indicate that UiO-66 NPs were successfully formed.

We evaluated the biocompatibility of UiO-66 NPs using a hemolysis assay. Even at a concentration of 400 µg mL^−1^, the hemolysis rate of UiO-66 NPs was < 5%, indicating their good biocompatibility (Additional file 1: Fig. S2A). Moreover, the results of the MTT assay showed that UiO-66 NPs had no toxic effect in A549, MDCK, and RAW264.7 cells (37 ℃, 48 h) at concentrations up to 1600 μg mL^−1^ (Additional file 1: Fig. S2B). No significant difference in body weight was observed among groups (Additional file 1: Fig. S2C). Inductively coupled plasma optical emission spectrometry was used to determine the biodistribution of Zr^4+^. The level of Zr^4+^ retained in all the organs was higher in the treated group than in the PBS group. Compared to the intranasal treatment group, the Zr^4+^ content in the liver, spleen, and kidney of the intraperitoneal injection group was significantly increased, whereas it was significantly decreased in the lungs and trachea (Additional file 1: Fig. S2D).

We assessed the biosafety of UiO-66 NPs in mice via intraperitoneal and intranasal administration of 50 µL of a UiO-66 NP 5% glucose suspension (80 mg kg^−1^). The control group was administered an equal volume of 5% glucose solution. Blood samples were collected for biochemical analysis on 14 d following administration. Hematological and biochemical analyses of the blood showed no differences in routine blood test results or liver and kidney functions among the three groups (Additional file 1: Fig. S2E, F). The levels of these indicators in the treatment groups were consistent with those in healthy mice. Thus, we confirmed that UiO-66 NPs were safe for animals at the doses evaluated. Organs, including the heart, liver, spleen, lungs, kidneys, brain, nose, and trachea, were collected on14 d following administration. The histopathological results indicated that neither treatment caused any damage or toxicity to the normal structure of the major organs in the mice (Additional file 1: Fig. S3). Collectively, these in vivo data suggest that UiO-66 NPs are highly biocompatible and safe for clinical use in intranasal and intraperitoneal delivery.

### Antiviral activity of UiO-66 NPs in vitro

**Fig. 2:**
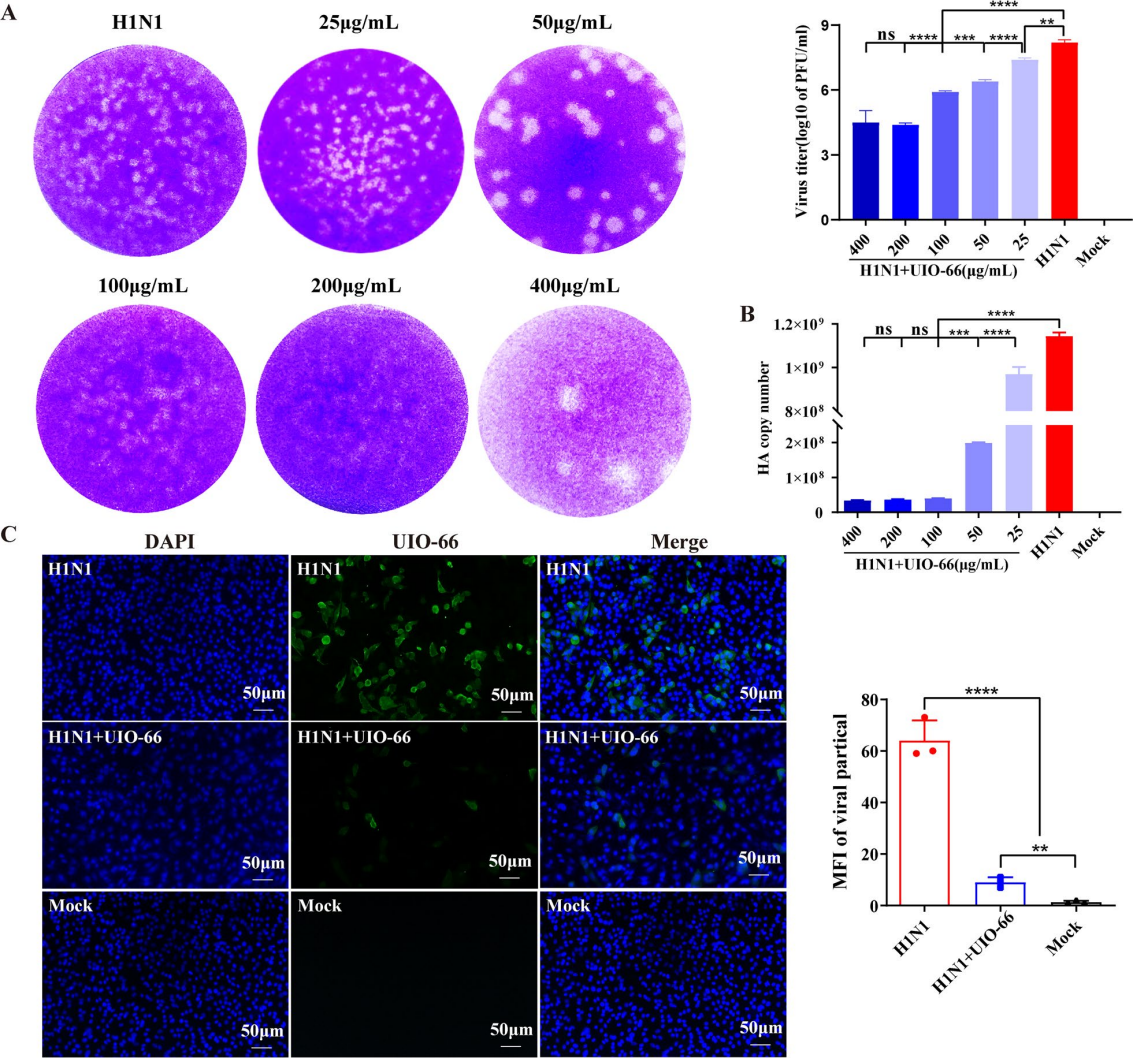
Antiviral activity of UiO-66 NPs in vitro. **A** Extracellular IAV treated with UiO-66 NPs detected by plaque assay. ns, *P* ≥ 0.05, **P < 0.01, ***P < 0.001, ****P < 0.0001. **B** Analysis of HA gene replication after IAV infection treated with UiO-66 NPs via qPCR. ns, *P* ≥ 0.05, ***P < 0.001, ****P < 0.0001. **C** Immunofluorescence detected IAV nucleoprotein (in green) in A549 cells at 24 h post-infection. **P < 0.01, ****P < 0.0001

To evaluate the anti-H1N1 effect of UiO-66 NPs in A549 cells, we collected the A549 cell supernatant at 24 h and detected the virus titer using a plaque assay. The virus titer of each group treated with UiO-66 NPs (25, 50, 100, 200, and 400 μg mL^−1^) was lower than that of the IAV group, and the lowest and optimal antiviral concentration was 200 μg mL^−1^ UiO-66 NPs (Fig. 2A). The viral hemagglutinin (HA) gene copy number in each group treated with UiO-66 NPs (25, 50, 100, 200, and 400 μg mL^−1^) was lower than that in the IAV group, as determined using qPCR. The optimal HA gene replication inhibition and lowest concentration was 100 μg mL^−1^ UiO-66 NPs (Fig. 2B), which indicates that UiO-66 NPs exhibited a concentration-dependent antiviral activity at an optimal dose of 200 μg mL^−1^. While, ZrCl_4_ or TPA incubation did not affected viral HA gene replication (Additional file 1: Fig. S4A–D). Finally, H1N1-infected cells were treated with 200 μg mL^−1^ UiO-66 NPs, and the intracellular viral nucleoprotein was analyzed using immunofluorescence staining. Compared with the IAV group, cells treated with UiO-66 NPs exhibited less green-positive signals (Fig. 2C).

**Fig. 3:**
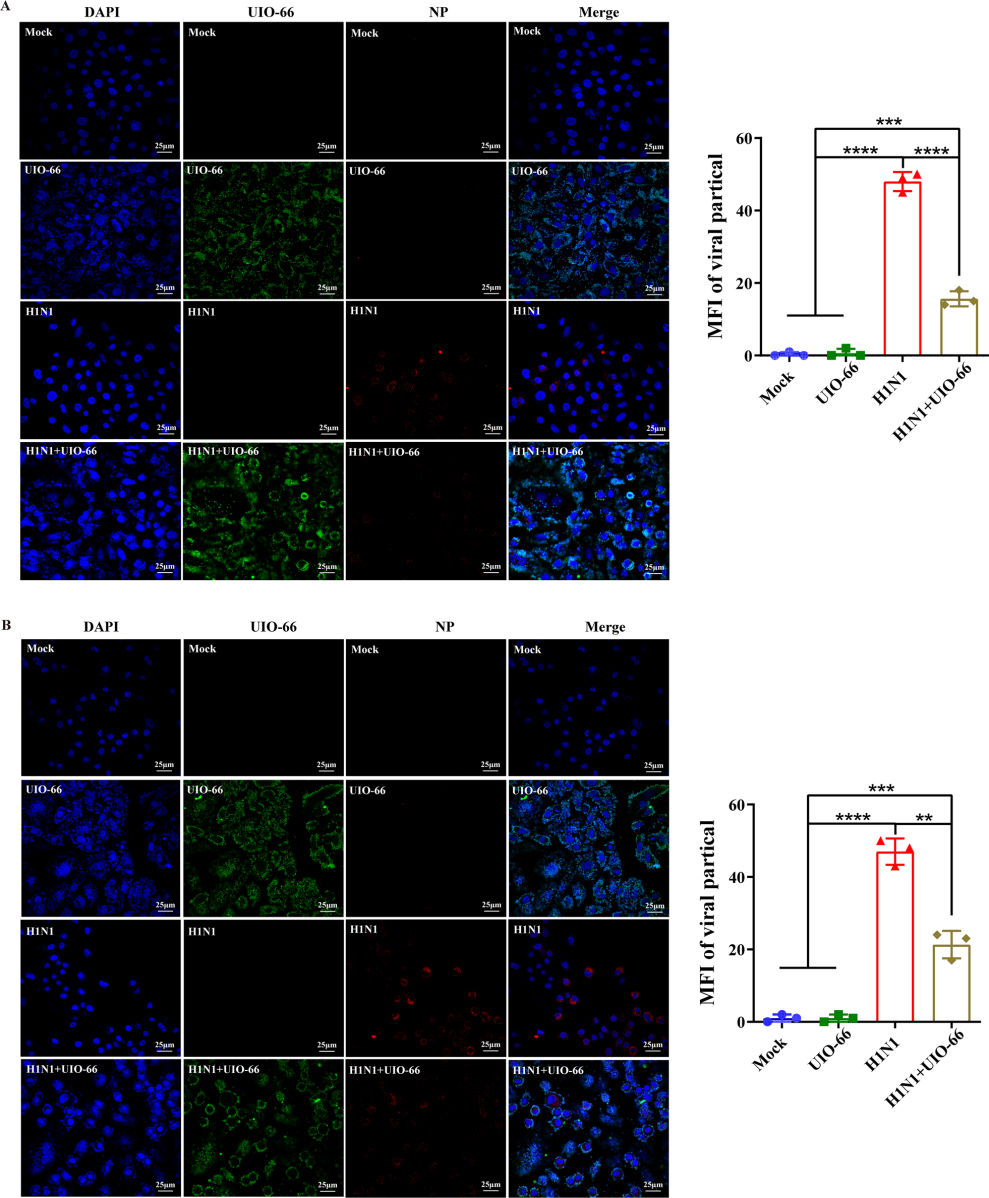

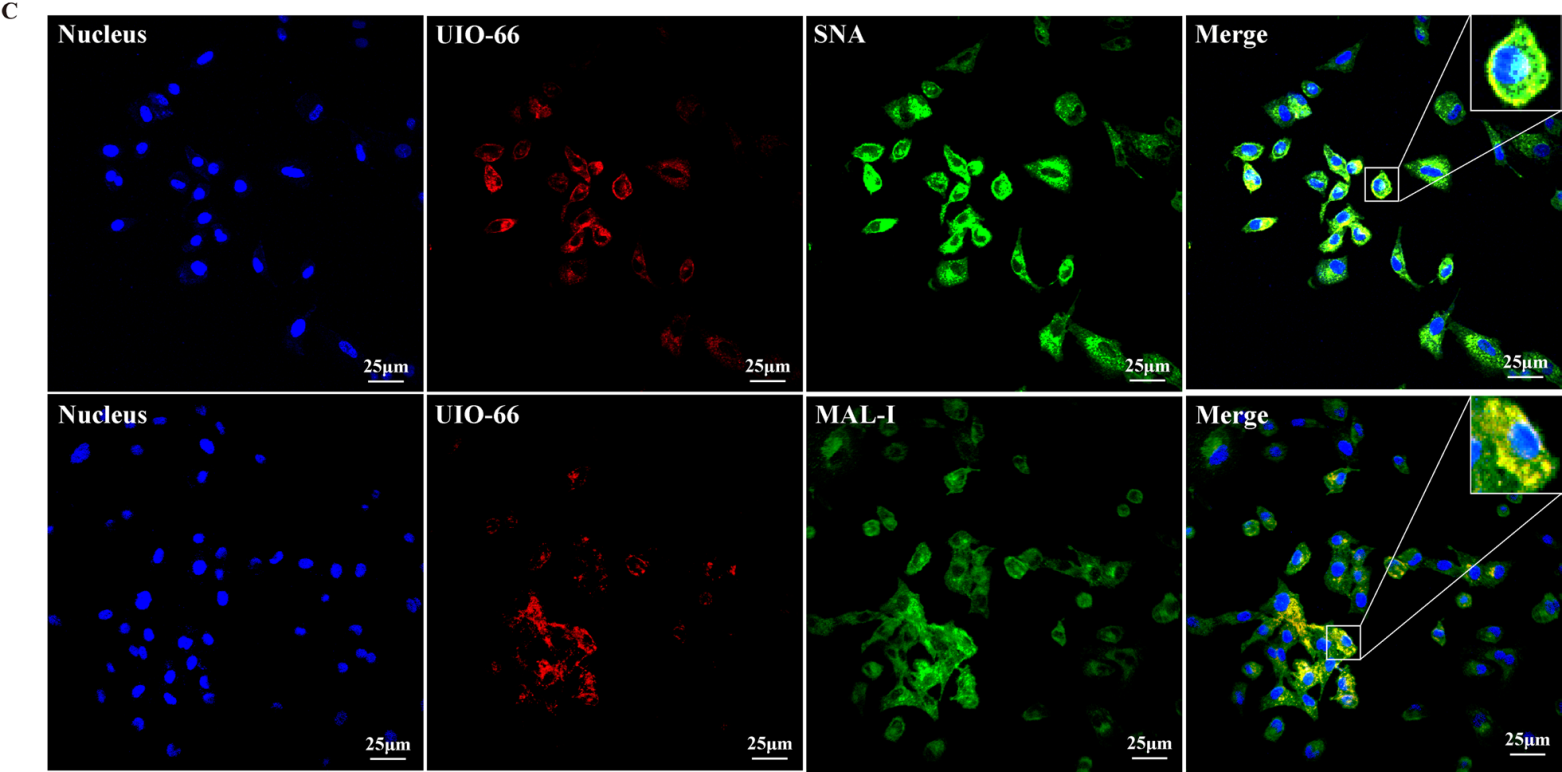
Inhibition of UiO-66 NPs on IAV proliferation. Immunofluorescence detected the effect of UiO-66 NPs (in green) to the infectivity of A549 cells on the **A** adsorption, **B** invasion processes of IAV (nucleoprotein, in red). **P < 0.01, ***P < 0.001, ****P < 0.0001. **C** Lectin histochemistry detected the colocalization (in yellow) of UIO-66 (in red) and sialic acid receptor (in green) in A549 cells

The potential antiviral mechanism of UiO-66 NPs (200 μg mL^−1^) was explored by analyzing the adsorption and invasion of UiO-66 NPs in IAV on their antiviral properties. We first tested whether UiO-66 NPs could directly inactivate IAV. As shown in Additional file 1: Fig. S5, UiO-66 NPs do not reduce the viral titer of IAV in the plaque assay, indicating that UiO-66 NPs cannot directly inactivate IAV. During IAV adsorption, confocal images showed that the experimental group had a significant inhibitory effect on IAV adsorption (Fig. 3A). During IAV invasion, treatment with UiO-66 NPs reduced intracellular invasion of the virions (Fig. 3B). To further observe the effect of UiO-66 NPs on viral adsorption, UiO-66 NPs (Cy3-labeled) and sialic acid receptors (FITC-labeled SNA or MAL-I) on the cell membrane were observed. The results showed that there was a large amount of co-localization of UIO66 and SNA (or MAL-I) (Fig. 3C), which indicated that the UIO-66 NPs probably could block the sialic-acid receptors for inhibiting influenza virus binding to the cell membrane. Therefore, these results suggest that UiO-66 NPs inhibited IAV primarily by inhibiting IAV adsorption and the invasion of host cells.

### UiO-66 NP treatment activated the RIG-I-like receptor signaling pathway in A549 cells

**Fig. 4:**
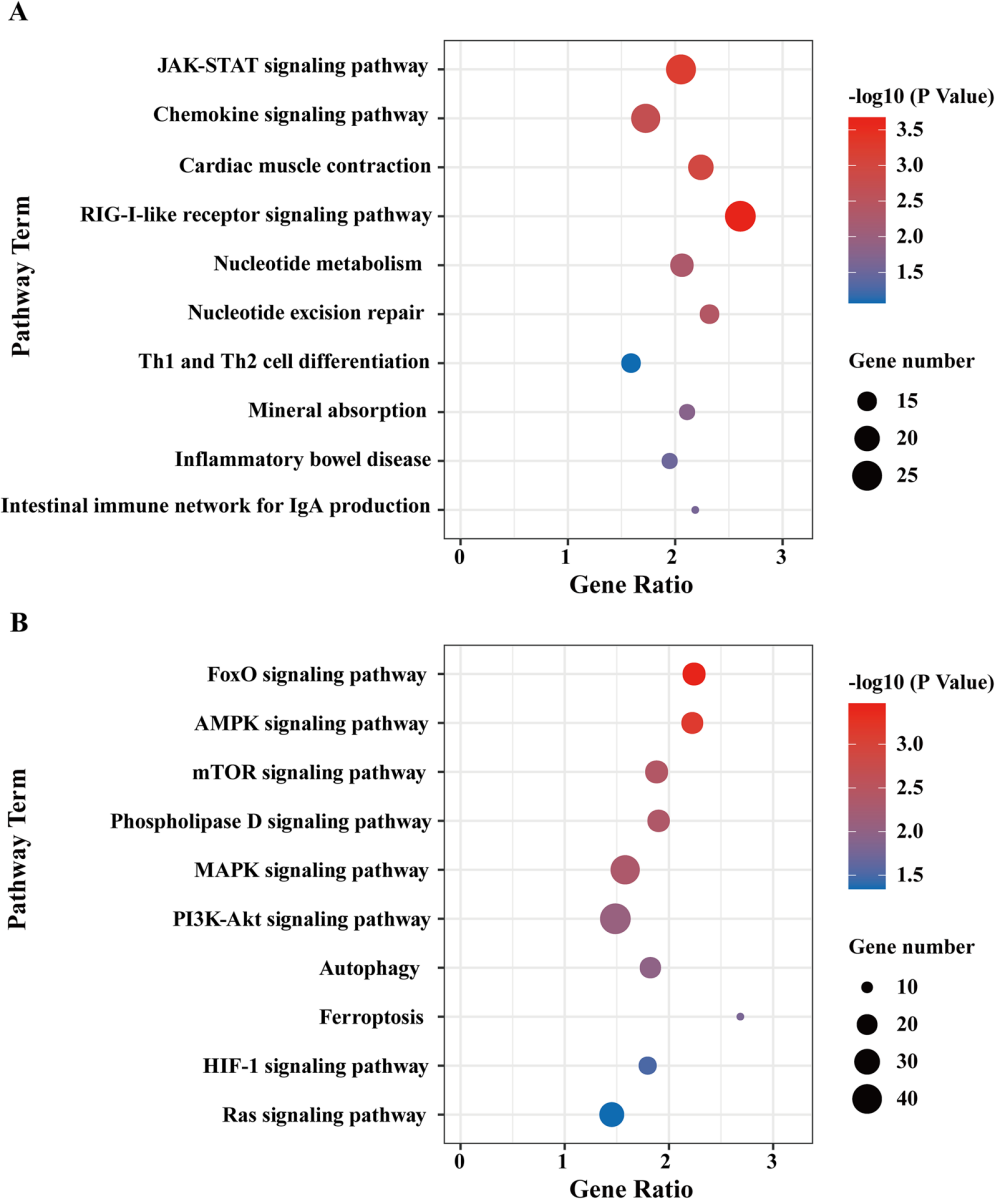
KEGG enrichment analysis of UiO-66 NPs treatment on IAV infected A549 cells. **A** Top 10 significant KEGG pathways associated with up regulated DEGs were differentially enriched in IAV + UiO-66_vs_IAV in A549 cells. **B** Top 10 significant KEGG pathways associated with down regulated DEGs were differentially enriched in IAV + UiO-66_vs_IAV in A549 cells

To further investigate the molecular mechanism of the anti-IAV effect of UiO-66 NPs in A549 cells, RNA-Seq analysis was performed. As shown in Additional file 1: Fig. S6A, 5251, 14, and 4121 differentially expressed genes (DEGs) were enriched in IAV_vs_Mock, UiO-66_vs_Mock, and IAV + UiO-66_vs_Mock, respectively. IAV infection in A549 cells induced 3323 upregulated and 1928 downregulated DEGs compared with mock (Additional file 1: Fig. S6A, C) and 2054 downregulated and 1513 upregulated DEGs compared with UiO-66 NP treatment infected with IAV (Additional file 1: Fig. S6B, D). These results indicate that UiO-66 NP treatment significantly decreased the number of DEGs. KEGG pathway enrichment analysis showed that the RIG-I-like receptor, Th1 and Th2 cell differentiation, and JAK-STAT signaling pathways were significantly upregulated following treatment with UiO-66 NPs (Fig. 4A). The FOXO, mTOR, and autophagy pathways were downregulated (Fig. 4B). These data suggest that UiO-66 NPs exert antiviral effects by activating the RIG-I-like receptor signaling pathway and alleviating the inflammatory pathway caused by IAV infection.

**Fig. 5:**
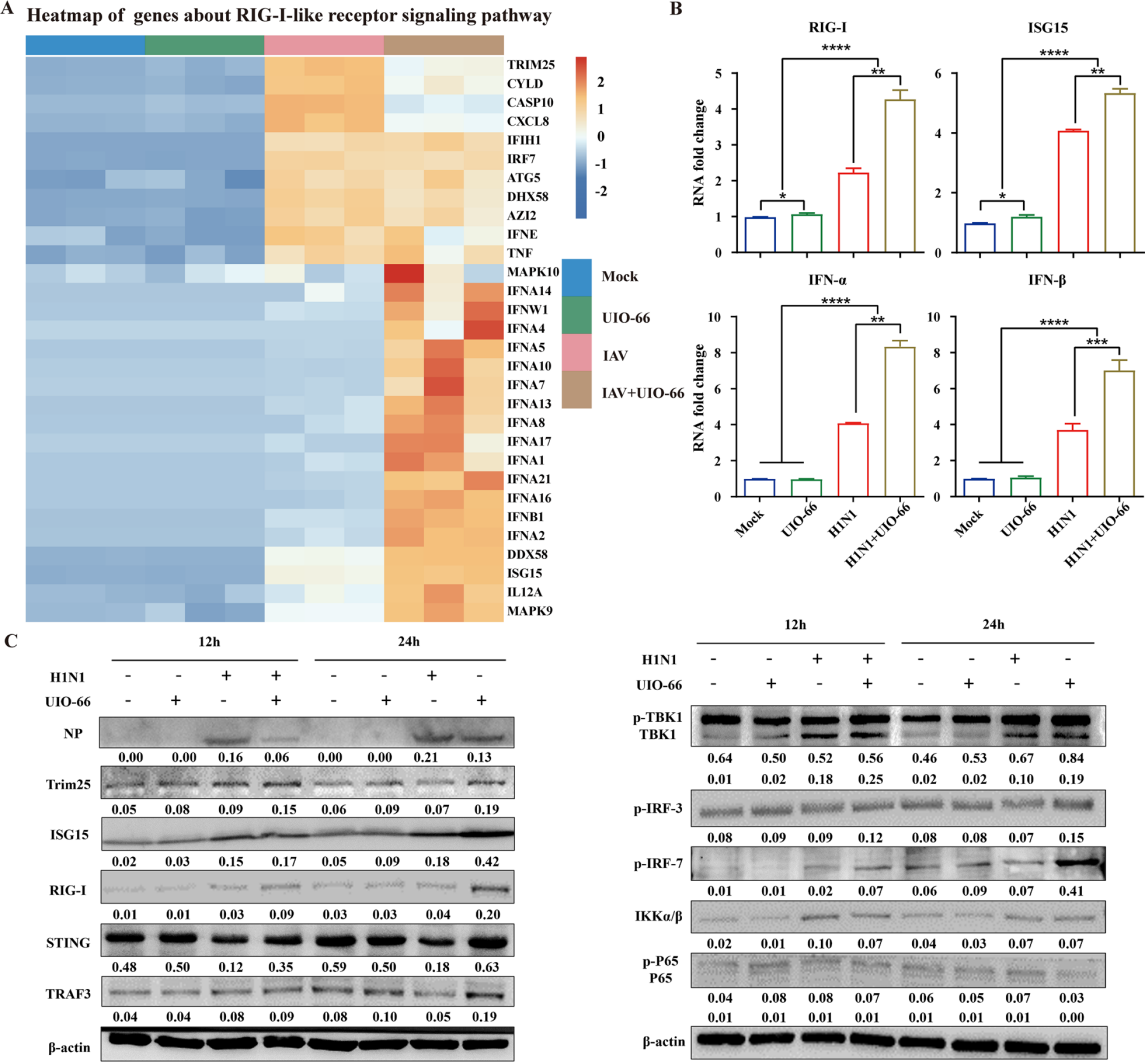
UiO-66 NPs treatment activated RIG-I-like receptor signaling pathway in A549 cells. **A** Heatmap of genes involved in RIG-I-like receptor signaling pathway. **B** Production of RIG-I, ISG15, IFN-α and IFN-β with the treatment of UiO-66 NPs using qPCR. **P < 0.01, ***P < 0.001, ****P < 0.0001. **C** Western blot detected related protein of RIG-I-like receptor signaling pathway in A549 cells at 12 h and 24 h post-infection

Heatmaps showed that UiO-66 NP treatment with IAV infection upregulated genes related to the RIG-I-like receptor signaling pathway (Fig. 5A). qPCR was used to detect the expression of major genes related to the RIG-I-like receptor signaling pathway. The results showed that the mRNA expressions of RIG-I, ISG15, IFN-α, and IFN-β were significantly higher following treatment with UiO-66 NPs (24 h, 37 ℃) compared with the IAV infected group (Fig. 5B), which was consistent with the transcriptomic results. Western blot analysis showed that UiO-66 NP treatment increased the expressions of upstream Trim25, RIG-I, and STING and downstream p-IRF3/7 in the RIG-I-like receptor signaling pathway, but did not induce changes in IKK-α/β and p-P65. Moreover, nucleoprotein expression of the virus was reduced following UiO-66 NP treatment (12 h or 24 h, 37 ℃), which was consistent with the qPCR results (Fig. 5C).

**Fig. 6:**
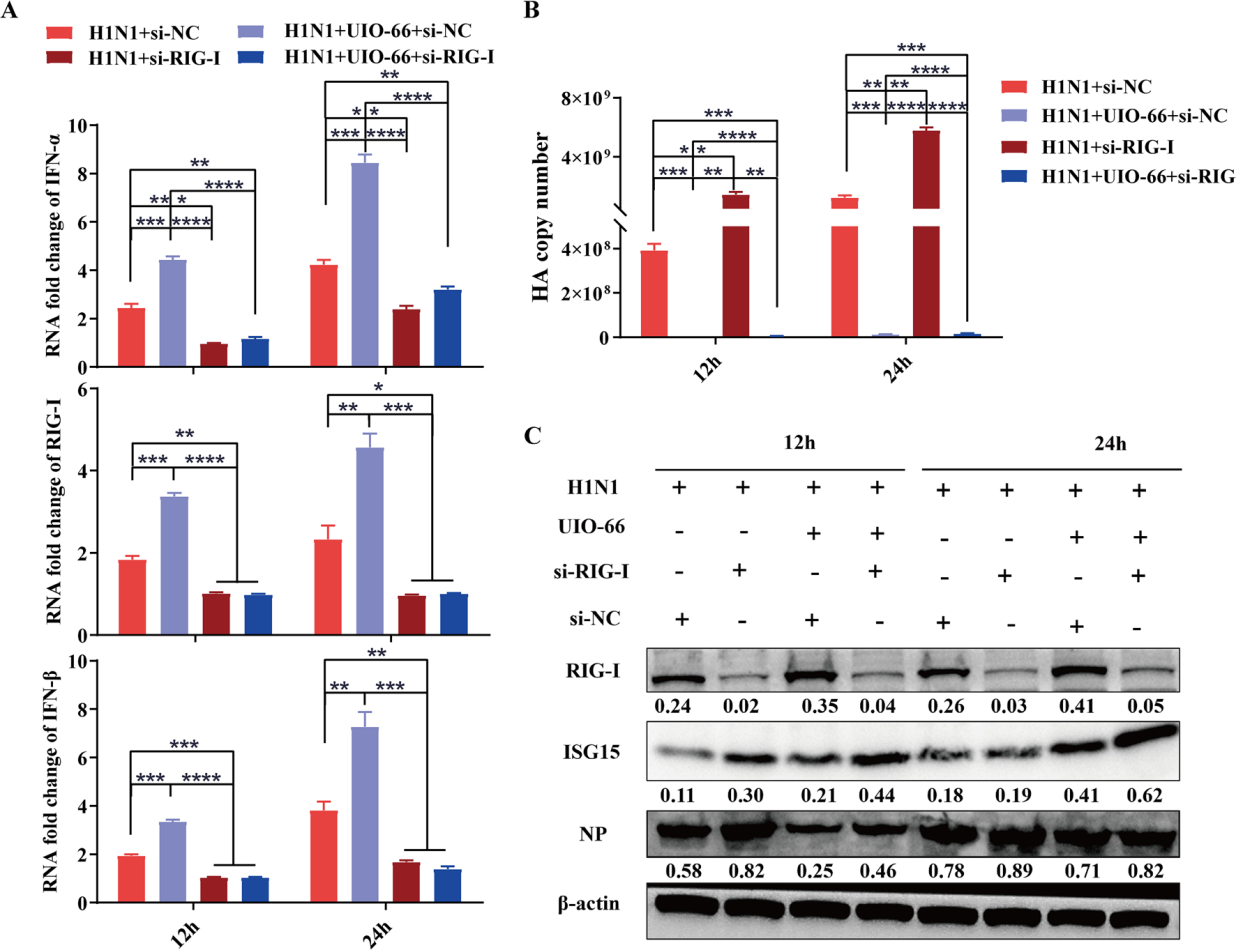
RIG-I siRNA transfection reversed the activation of RIG-I-like receptor signaling pathway caused by UiO-66 treatment in A549 cells. **A** Production of RIG-I, IFN-α, and IFN-β after siRNA interference in A549 cells at 12 h and 24 h post-infection via qPCR. *P < 0.05, **P < 0.01, ***P < 0.001, ****P < 0.0001. **B** HA gene copy number of IAV in A549 estimated using real-time PCR. **P < 0.01, ***P < 0.001, ****P < 0.0001. **C** Western blot detected RIG-I, ISG15 and nucleoprotein in A549 cells after siRNA transfection at 12 h and 24 h post-infection

To further confirm the relationship between the RIG-I-like receptor signaling pathway and UiO-66 NPs, si-RIG-I or si-NC were transfected into A549 cells (37 ℃, 8 h). First, after si-RIG-I gene interference, changes in the downstream genes of the RIG-I-like receptor pathway and viral HA mRNA were detected using qPCR following UiO-66 NP treatment (12 h or 24 h, 37 ℃). As shown in Fig. 6A, B, compared with A549 cells transfected with si-NC, the mRNA expression levels of RIG-I, IFN-α, and IFN-β are significantly lower in A549 cells transfected with si-RIG-I, while the copy number of HA is significantly higher. Next, the viral load was determined using western blotting. Western blot results showed that the protein expression of viral nucleoproteins that were reduced by the UiO-66 NP treatment was reversed in si-RIG-I-treated A549 cells (Fig. 6C).

**Fig. 7:**
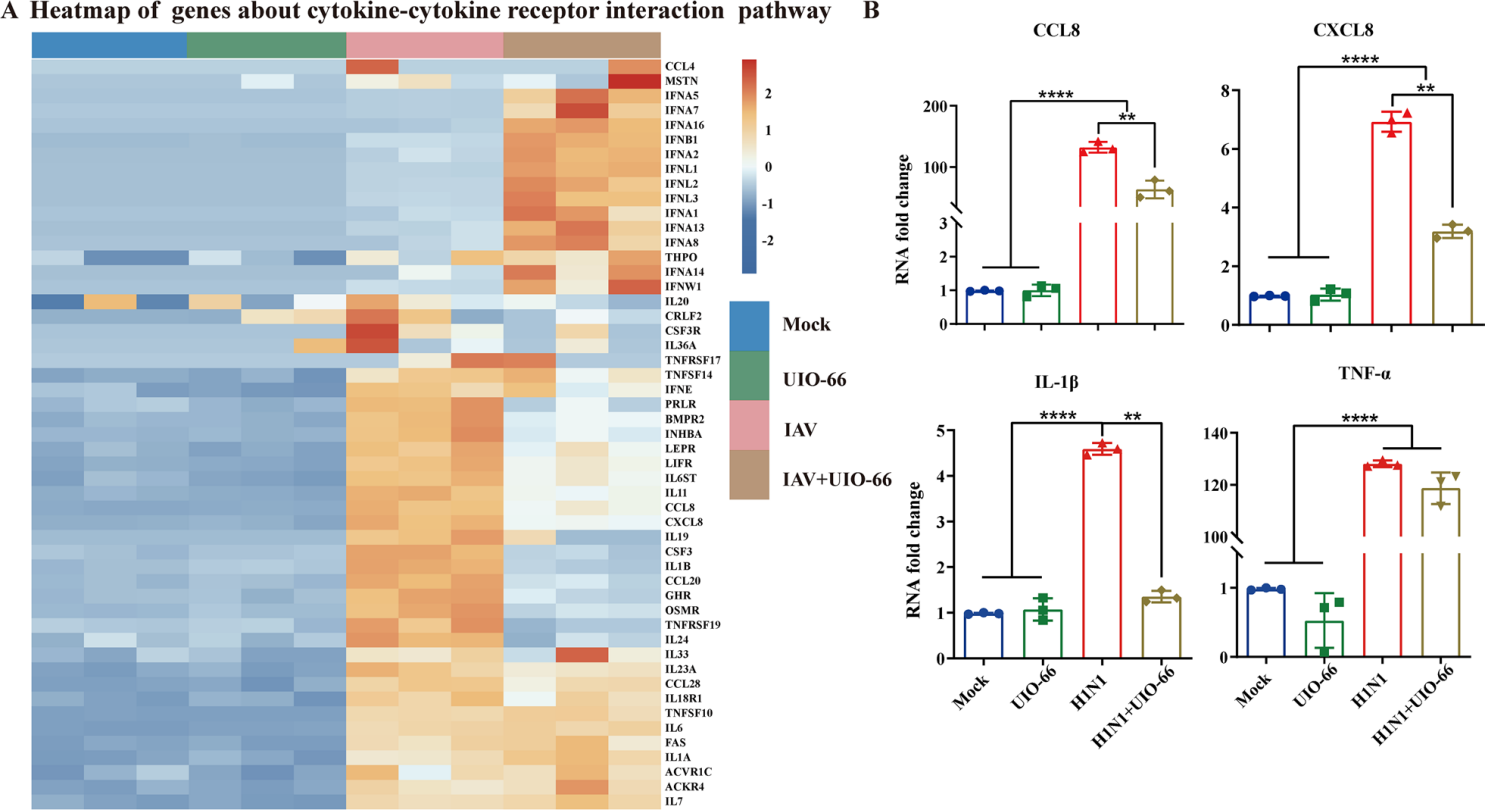
UiO-66 NPs treatment inhibited the production of cytokines and chemokines of A549 cells. **A** Heatmap of genes about cytokine-cytokine receptor interaction pathway. **B** Production of CCL8, CXCL8, IL-1β and TNF-α with the treatment of UiO-66 NPs using qPCR. **P < 0.01, ****P < 0.0001

To further investigate whether UiO-66 NP treatment can affect the expression of cytokines induced by IAV infection, we analyzed the genes associated with the cytokine–cytokine receptor interaction pathway (Fig. 7A). Heat maps showed that the UiO-66 NP treatment of IAV-infected A549 cells significantly downregulated certain genes related to the cytokine–cytokine receptor interaction pathway. We subsequently examined the effects of the relevant cytokines and chemokines using qPCR. As shown in Fig. 7B, UiO-66 NPs significantly downregulated the levels of CCL-8, CXCL-8, IL-1β, and TNF-α in A549 cells at 24 h post IAV infection. These data suggest that treatment with UiO-66 NPs reduced the release of inflammatory factors caused by IAV infection.

### UiO-66 NP treatment reduced acute lung damage caused by IAV infection

**Fig. 8:**
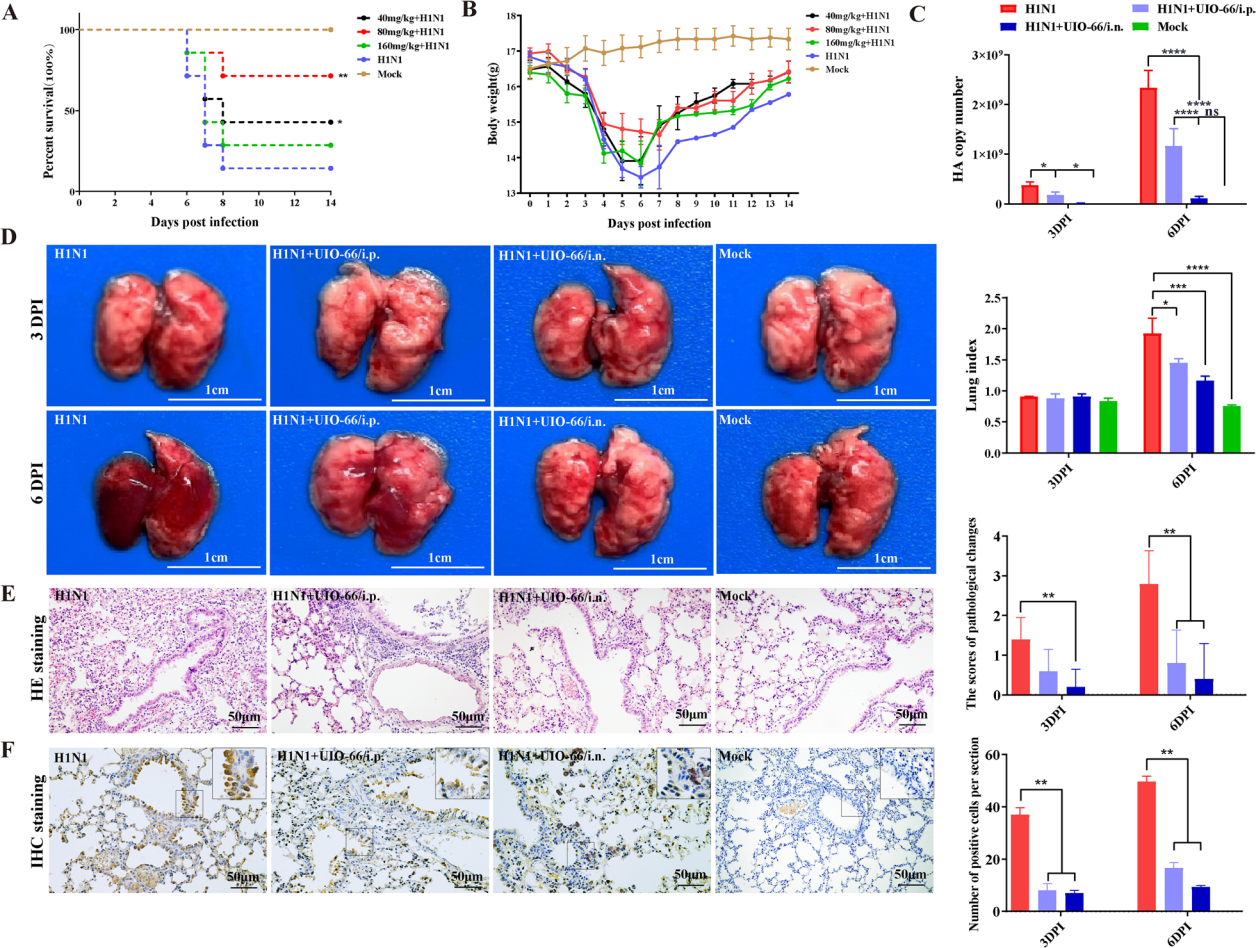
UiO-66 NPs treatment reduced acute lung damage caused by IAV infection. **A** Survival rates (Compared with the H1N1 group, log-ranked Mantel Cox test, *P < 0.05, **P < 0. 01) and **B** body weight changes of mice (UiO-66 NPs, i.n.) (n = 7/group). **C** HA gene copy number of IAV in lung tissues estimated using qPCR. Data are representative of three independent experiments. *P < 0.05, ****P < 0.0001. **D** Lung tissue images and lung index analysis after the UiO-66 NPs treatment of IAV infection. *P < 0.05, ***P < 0.001, ****P < 0.0001. **E** Representative images of histopathologic changes of lung tissues stained with hematoxylin and eosin. **P < 0.01. **F** Immunohistochemistry staining of viral nuclear proteins in lung tissues of mice infected with H1N1 treated with UiO-66 NPs. **P < 0.01

Intranasal administration was used to further investigate the protective effect of UiO-66 NPs against H1N1 viral infection in mice. First, the H1N1-infected mice were treated with three doses of UiO-66 NPs. The survival rates and body weights of the mice were recorded after 14 d. As shown in Fig. 8A, at 8 DPI, the survival rate of the H1N1 virus challenge group was 14.3% (1/7), while those of the 40 and 80 mg kg^−1^ UiO-66 NP treatment groups were 71.4% (5/7) and 42.8% (3/7), respectively, which were significantly higher than those of the IAV group. The weight loss trend was similar in the H1N1 virus challenge and the UiO-66 NP treatment groups. Compared with the H1N1 virus challenge group, the UiO-66 NP treatment group showed slightly less weight loss from 2 to 7 DPI. At 7 DPI, as the surviving UiO-66 NP-treated mice increased in weight, the weight recovery of the H1N1 virus-challenged group was lower than that of the treatment group. No significant clinical signs, weight loss, or death occurred in control mice (Fig. 8B). These results suggest that UiO-66 NP treatment can partially ameliorate weight loss and improve the survival rate of mice infected with the H1N1 virus.

We investigated whether UiO-66 NPs were directly involved in anti-viral infection using both intraperitoneal and intranasal approaches. Lung lesions from three mice of each group were observed at 3 and 6 d following infection (Fig. 8D). On 6 DPI, the lung index was significantly lower in the UiO-66-treated group than in the IAV group. Severe bronchitis was observed in the lungs of mice infected with IAV on 6 DPI, which was characterized by excessive bleeding and severely damaged alveolar cavities and inflammatory cells. In contrast, abdominal injection in the dosage group resulted in lighter lesions, including numerous inflammatory cell infiltration around the bronchioles. Notably, only minor damage was observed among mice in the UiO-66 NP intranasal treatment group (Fig. 8E).

As shown in Fig. 8F, high levels of nucleoprotein antigens are expressed in the bronchial epithelial cells of mice infected with the H1N1 virus, whereas no positive signal is observed in the bronchial epithelial cells of mice treated with UiO-66 NPs intranasally. Moreover, a minimal positive signal was observed in the bronchial epithelial cells of mice treated with a UiO-66 NP intraperitoneal injection. No positive signal was detected in the lungs of mice in the control group. At 6 DPI, the nucleoprotein antigen score in the lungs of the IAV group was significantly higher than that in the UiO-66 NP treatment group, and the nucleoprotein antigen score in the intranasal administration group was significantly higher than that in the intraperitoneal injection group.

Viral loads in the lung tissues of mice were detected using real-time PCR. The fold-change in HA gene levels in the lungs of mice treated with UiO-66 NPs was significantly lower than that of the H1N1 virus challenge group at 3 and 6 d following infection. The intranasal administration group was significantly lower than that in the intraperitoneal injection group (Fig. 8C). Therefore, our data suggest that UiO-66 NPs can exert anti-H1N1 virus effects in mice lungs.

**Fig. 9:**
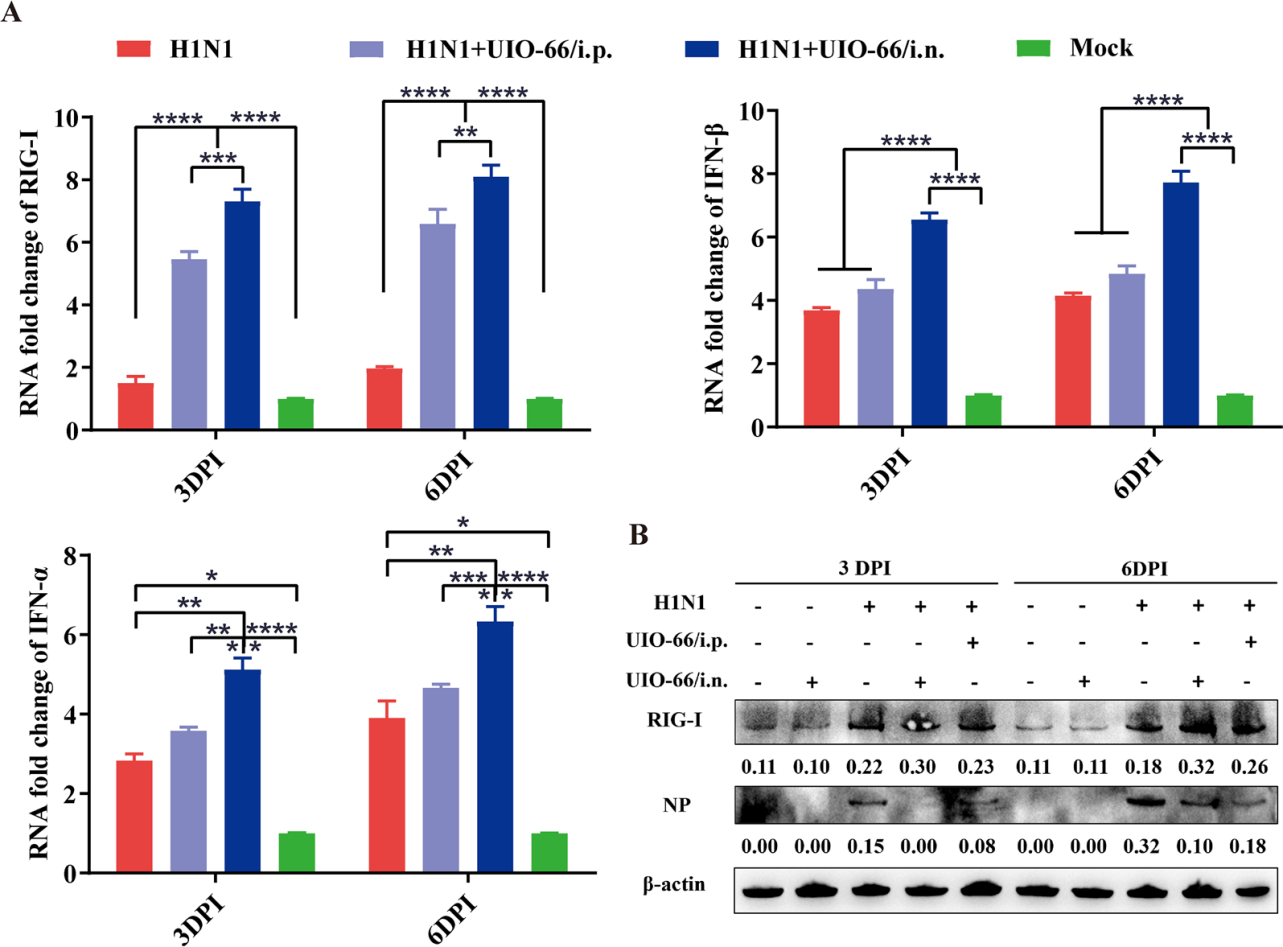
UiO-66 NPs treatment activated the RIG-I-like receptor signaling pathway in the lung tissue of mice. **A** Production of RIG-I, IFN-α and IFN-β with the treatment of UiO-66 NPs using qPCR. *P < 0.05, **P < 0.01, ***P < 0.001, ****P < 0.0001. **B** Western blot detected RIG-I and nucleoprotein in the lung tissue of mice

To further confirm whether UiO-66 NPs activated the RIG-I-like receptor signaling pathway in mice, we used qPCR to detect key genes, such as RIG-I, IFN-α, and IFN-β, in the pathway. The mRNA expressions of RIG-I, IFN-α, and IFN-β were significantly upregulated in the UiO-66 NP treatment group at 3 and 6 DPI, and the mRNA expressions of RIG-I, IFN-α, and IFN-β were higher in the intranasal injection group than in the intraperitoneal injection group (Fig. 9A). We examined the expressions of RIG-I and viral nucleoproteins using western blotting. We found that the RIG-I protein was significantly upregulated by treatment with UiO-66 NPs and was higher in the intranasal group than in the intraperitoneal group (Fig. 9B). Treatment with UiO-66 NPs significantly reduced the amount of nucleoprotein, and the effect of the intranasal injection was greater than that of the intraperitoneal injection.

## Discussion

IAV has caused seasonal influenza epidemics and pandemics, resulting in serious threats to public health and socioeconomic impacts [32]. Antigenic drift or shift to generate new variants results in declining vaccination effectiveness [33], and the continuous emergence of drug-resistant strains affects the application of drugs [16], so the emergence of new drugs is urgently needed. Research on nanomaterials has become an emerging hotspot, significantly expanding their applications [34]. With the development of metal nanomaterials in antitumor research, they have shown extensive application prospects in biological fields [35, 36]. In fact, metals NPs (including copper, silver, zirconium nano particles and its nano-complexes) have been demonstrated to have potential antiviral capabilities. For example, the antiviral effect of Cu-based nanomaterials is accomplished by autophagy, destruction of viral membrane and genomic material, interference for the essential viral protein, and killing of the virus by ROS generation as demonstrated for SARS-CoV-2 variants [37–39]. It has been reported that 160-nm CuI radicals effectively generated hydroxyl radicals to fight against the influenza virus [40]. The metal Ag-based NPs and its compounds were used to prevent viruses from entering host cells by blocking the active site or directly attached to the destruction of virus envelope and genomic substances [41]. Our previous study showed the antiviral potential to H5N1 infection in mice of Zirconium-based nanomaterials [30]. Furthermore, with the recent outbreak of the novel coronavirus, researchers are focusing on treating infectious viral diseases with metal-based nanomaterials [42–44].

UIO-66 NPs had no significant cytotoxicity effect on chondrocytes (up to 800 µg mL^−1^) and colon cancer cells (up to 1000 µg mL^−1^) [26, 27], which is consistent with the results of the present study that UiO-66 NPs (up to 1600 µg mL^−1^) in A549, MDCK and RAW267.4 cell has good biological safety. In this study, we screened UIO-66 nanomaterials with antiviral activity against the IAV and explored their possible inhibitory stages. As antiviral therapeutic materials against viral infection, the two inhibition pathways of metal nanomaterials, including the inhibition of virus adsorption and entry [45, 46] and intracellular virus suppression, are extremely common [47–49]. Our results showed that UiO-66 NPs nonspecifically shielded the viral sialic acid receptors on the cell membrane to combat virus. Further studies on the mechanism of UiO-66 NPs and their antiviral effects will identify related molecules and pathways.

The induction of IFN-based antiviral innate immunity is dependent on pattern recognition receptors, including TLRs and RIG-I-like receptors [50]. RIG-I is critical for activating type I IFN-dependent antiviral innate immune response, recognizes viral genomic RNA during negative-strand RNA virus infection [51], and triggers type I interferon-mediated immune responses to protect the host from viral infection. In this study, UiO-66 NPs, but not TPA or zirconium ion, reduced viral replication in A549 cells. RIG-I-like receptor signaling was identified as one of the major mechanisms underlying the antiviral effect of UiO-66 NPs using RNA-Seq analysis. We found that UiO-66 NPs significantly activated the expressions of RIG-I and ISG15 and enhanced the production of downstream type I interferons, thereby inhibiting viral replication in IAV-infected A549 cells. The knockdown of RIG-I by specific siRNAs significantly reduced the expressions of IFN-α and IFN-β cytokines induced by UiO-66 NPs. The copy number of viral HA gene and expression of nucleoprotein were also significantly increased. This indicates that the protective effect of UiO-66 NPs against IAV infection was dependent on the presence of RIG-I, which is a key receptor involved in viral infection [52]. In addition, RIG-I directly inhibits viral replication independent of antiviral signaling [53]. The present data showed that the JAK-STAT signaling pathway was upregulated by UiO-66 NPs treatment. Mahony et al. showed that the key molecule in the pathway, STAT3, shRNA knockdown increased PR8 IAV replication [54]. The JAK/STAT pathway is a classical antiviral signaling pathway, causing the generation of downstream antiviral IFN-stimulated genes (ISGs) [55]. 2′-5′-oligo-adenylate synthetase (OAS) and protein kinase R (PKR) are two families belonging to ISGs. OAS–RNase L system limited the replication of many different viruses, such as picornaviruses, flaviviruses and alphaviruses [56–59]. It has been found that NS3 of H5N2 viral protein enhanced viral replication and pathogenicity in mammalian systems potentially via suppression of PKR activity [60]. The IFN-induced transmembrane (IFITM) protein is the prominent antiviral ISG product, which could suppress the replication of H1N1-IAV [61]. Interferon-stimulated gene 15 (ISG15) is one of the most rapidly and robustly induced genes that respond to type I interferon stimulation [62, 63]. UiO-66 NPs can also induce the upregulation of ISG15 in the study, which may be the primary reason for its potential as a antiviral agent.

Our study showed that treatment with UiO-66 NPs significantly downregulated the HIF-1, MAPK, FOXO, mTOR and autophagy signaling pathways. IAV increased glycolysis to promote viral replication by inducing HIF-1 stabilization, transcription, translation, and activation [64]. MAPK and autophagy signaling pathways contributed to IAV virus-induced inflammation and lung injury [65, 66]. FOXO signaling pathway has also been showed playing an important role in the IAV-induced alveolar macrophage dysfunction [67], and FoxO1 negatively regulated cellular antiviral response by promoting degradation of IRF3 [68]. The mTOR signaling pathway was most important for early IAV replication, and the use of mTOR inhibitor rapamycin could block IAV replication in vitro [69, 70]. Moreover, IAV infection can produce various cytokines and chemokines that trigger the activation of nuclear factor κb (NF-κB) and induce proinflammatory cytokines and chemokines, such as IL-6 and TNF-α [71]. The over-production of proinflammatory cytokines and overactivation of immune cells during IAV infection is known as a cytokine storm. Using qPCR, we confirmed that UiO-66 NP treatment reduced the production of IL-1β, TNF-α, CCL8, and CXCL8 in IAV infection. In addition, the expressions of p65 and IKKα/β, the key proteins in the NF-κB signaling pathway, were slightly reduced. Therefore, our results suggest that UiO-66 NP treatment did not excessively activate the NF-κB inflammatory signaling pathway, but instead decreased inflammation while enhancing the type I interferon signaling pathway. Our previous study showed that zirconia-based nanoparticles (ZrO_2_) at the dose of 50 mg kg^−1^ and 100 mg kg^−1^ could reduce the overexpression of pro-inflammatory cytokines induced by IAV-H5N1 to alleviate mouse lung injury [30].

Subsequently, the efficacy of UiO-66 NP treatment was demonstrated in a mouse model, in which RIG-I expression was up-regulated, which was consistent with the in *vitro* results. Animals treated with UiO-66 NPs had a significantly faster recovery, significantly lower IAV load in the lungs, and increased IFN-stimulated gene expression, highlighting the induction of RIG-I. We also found a significant increase in the expressions of either IFN-α or IFN-β in lung tissue treated with UiO-66 NPs. Increased RIG-I expression activates the type I interferon and its downstream antiviral immune responses [72]. Our in vivo study showed that Uio66 NPs could reduce viral replication and load in lung tissue by activating the RIG-I-like receptor signaling pathway, which alleviated lung injury caused by IAV infection.

## Conclusion

Herein, we confirmed the antiviral effects and anti-inflammatory properties of UiO-66 NPs without significant toxicity in vitro or in vivo. UiO-66 NPs demonstrated excellent inhibition of the IAV. UiO-66 NPs primarily inhibited influenza virus cellular internalization and intracellular replication. In addition, UiO-66 NPs activated the RIG-I-like receptor signaling pathway, thereby enhancing the downstream type I interferon signaling pathway to exert antiviral effects. UiO-66 NPs alleviated inflammation by reducing the cytokine storm induced by IAV infection. Our results may shed light on a new, effective, and low-toxicity pandemic treatment strategy and provide a potential treatment for general hyperinflammation that is easily manufactured. Therefore, with further research, the application potential of zirconium-based nanomaterials in antiviral therapy should be further demonstrated.

### Supplementary Information

**Additional file 1:** **Table S1.** Primers of housekeeping gene β-actin and other targeted genes. **Figure S1.** **A** Hydrodynamic size of UiO-66 NPs in water. **B** Zeta potentials of UiO-66 NPs in water. **Figure S2.** The evaluation of UiO-66 NPs biocompatibility in vivo and in vitro (UiO-66 NPs, i.n. or i.p.). **A** Hemolysis rates of UiO-66 NPs. **B** Cell viability (MTT) of RAW264.7 cells, A549 cells and MDCK cells incubated with UiO-66 NPs for 48 h. **C** body weight changes of mice (n = 5/group). **D** Quantitative distribution analysis of Zr^4+^ in heart, liver, spleen, lung, kidney, brain, trachea and blood samples after treatment with UiO-66 NPs for 14 days in mice. **E** Serum biochemical indicators of in mice treated with UiO-66 NPs. **F** Hematological analysis of the mice treated with UiO-66 NPs. **Figure S3.** Representative hematoxylin and eosin staining of vital organs (heart, liver, spleen, lung, kidney, brain, nose and trachea) at 14 days after treated with UiO-66 NPs. **Figure S4.** The evaluation of toxicity and effect on HA gene replication of ZrCl4 or TPA in A549 cells. Cell viability (MTT) of A549 cells incubated with **A** ZrCl_4_ and **B** TPA for 48 h at 37 ℃. Analysis of HA replication after IAV infection treated with **C** ZrCl_4_ and **D** TPA at 24 h. **Figure S5.** Viral titers determined by plaque assay after 2 hours of incubation with UiO-66 NPs. ns, *P* ≥ 0.05. **Figure S6.** UiO-66 NPs could decrease the expression of DEGs in A549 cells infected with IAV. **A** Venn plots of DEGs from IAV+UiO-66 _vs_ Mock, IAV_vs_Mock and UiO-66 _vs_Mock. **B** Venn plots of DEGs from IAV_vs_UiO-66 , IAV+UiO-66 _vs_IAV and IAV+UiO-66 _vs_UiO-66. **C** Volcano plots of DEGs from IAV_vs_Mock. **D** Volcano plots of DEGs from IAV+UiO-66 _vs_ IAV.

## Data Availability

All data generated or analyzed during the study period are included in the paper. Additional data relevant to this study are available upon request.
