# Supplementary material for: UiO-66 nanoparticles combat influenza A virus in mice by activating the RIG-I-like receptor signaling pathway
Source: J Nanobiotechnology. 2024 Mar 9;22:99. doi: 10.1186/s12951-024-02358-y (PMC10925002; doi:10.1186/s12951-024-02358-y)

**Additional file 1**

**UiO-66 nanoparticles combat influenza A virus by activating the RIG-I-like receptor signaling pathway**

Ruijing Su^a^, Xinsen Li^a^, Jin Xiao^b^, Jiawei Xu^a^, Jijing Tian^a^, Tianlong Liu^a^*, Yanxin Hu^a^*

^a^*National Key Laboratory of Veterinary Public Health and Safety, Key Laboratory of Animal Epidemiology of Ministry of Agriculture and Rural Affairs, College of Veterinary Medicine, China Agricultural University, No. 2 Yuanmingyuan West Road, Beijing 100193, China;*

^b^*Key Laboratory of Veterinary Bioproduction and Chemical Medicine of the Ministry of Agriculture, Zhongmu Institutes of China Animal Husbandry Industry Co., Ltd, Beijing, People’s Republic of China.*

Tel: +86 010-62733398; +86 010 62731977

* Correspondences: [liutianlong@cau.edu.cn](mailto:liutianlong@cau.edu.cn); [huyx@cau.edu.cn](mailto:huyx@cau.edu.cn)

**Table S1.** Primers of housekeeping gene β-actin and other targeted genes

| **Target gene** | **Primer pairs (5'→3')** | |
| --- | --- | --- |
| β-actin-mouse | | F: 5’ - GAGACCTTCAACACCCCGC- 3’ |
|  |  | R: 5’ - ATGTCACGCACGATTTCCC- 3’ |
| RIG-I-mouse | | F: 5’ - ATTGTCGGCGTCCACAAAG- 3’ |
|  |  | R: 5’ - GTGCATCGTTGTATTTCCGCA-3’ |
| ISG15-mouse | | F: 5’ - CAG​GAC​GGT​CTT​ACC​CTT​TCC- 3’ |
|  |  | R: 5’ - AGG​CTC​GCT​GCA​GTT​CTG​TAC-3’ |
| IFN-α-mouse | | F: 5’ - AGCCTTGACACTCCTGGTACA- 3’ |
|  |  | R: 5’ - TGAGCCTTCTTGATCTGCTG-3’ |
| IFN-β-mouse | | F: 5’ - AGATGTCCTCAACTGCTCTC- 3’ |
|  |  | R: 5’ - AGATTCACTACCAGTCCCAG-3’ |
| β-actin-human | | F: 5’ - CATGTACGTTGCTATCCAGGC - 3’ |
|  |  | R: 5’ - CTCCTTAATGTCACGCACGAT - 3’ |
| IL-1β-human | | F: 5’ - GTGGCAATGAGGATGACTTGTTC - 3’ |
|  |  | R: 5’ - TAGTCGGTGGTCGGAGATTCGTA - 3’ |
| TNF-α-human | | F: 5’ - TCCCCAGGGACCTCTCTCTA - 3’ |
|  |  | R: 5’ - AGGGTTTGCTACAACATGGGC -3’ |
| CCL8-human | | F: 5’ - ACTTGCTCAGCCAGATTCAGTT - 3’ |
|  |  | R: 5’ - GACCCATCTCTCCTTGGGGT -3’ |
| CXCL8-human | | F: 5’ - GTTGTAGCGTAGCTAGATGC- 3’ |
|  |  | R: 5’ - TTCTCGTGTGCAGTATCTACG-3’ |
| RIG-I-human | | F: 5’ - TCCTTTATGAGTATGTGGGCA- 3’ |
|  |  | R: 5’ - TCGGGCACAGAATCTTTG-3’ |
| ISG15-human | | F: 5’ - CTCTGAGCATCCTGGTGAGGAA- 3’ |
|  |  | R: 5’ - AAGGTCAGCCAGAACAGGTCGT-3’ |
| IFN-α-human | | F: 5’ - TGGGCTGTGATCTGCCTCAAAC- 3’ |
|  |  | R: 5’ - CAGCCTTTTGGAACTGGTTGCC-3’ |
| IFN-β-human | | F: 5’ - GCTCTCCTGTTGTGCTTCTCCAC- 3’ |
|  |  | R: 5’ - CAATAGTCTCATTCCAGCCAGTGC-3’ |
| H1N1-HA | | F: 5’- CGCAGTATTCAGAAGAAGCAAGAC-3’ |
|  |  | R: 5’- TCCATAAGGATAGACCAGCTACCA-3’ |

Figure S1. (A) Hydrodynamic size of UiO-66 NPs in water. (B) Zeta potentials of UiO-66 NPs in water.

Figure S2. The evaluation of UiO-66 NPs biocompatibility in *vivo* and in *vitro* (UiO-66 NPs, i.n. or i.p.). (A) Hemolysis rates of UiO-66 NPs. (B) Cell viability (MTT) of RAW264.7 cells, A549 cells and MDCK cells incubated with UiO-66 NPs for 48 h. (C) body weight changes of mice (n=5/group). (D) Quantitative distribution analysis of Zr^4+^ in heart, liver, spleen, lung, kidney, brain, trachea and blood samples after treatment with UiO-66 NPs for 14 days in mice. (E) Serum biochemical indicators of in mice treated with UiO-66 NPs. (F) Hematological analysis of the mice treated with UiO-66 NPs.

Figure S3. Representative hematoxylin and eosin staining of vital organs (heart, liver, spleen, lung, kidney, brain, nose and trachea) at 14 days after treated with UiO-66 NPs.

Figure S4. The evaluation of toxicity and effect on HA gene replication of ZrCl4 or TPA in A549 cells. Cell viability (MTT) of A549 cells incubated with (A) ZrCl_4_ and(B) TPA for 48 h at 37 ℃. Analysis of HA replication after IAV infection treated with (C) ZrCl_4_ and(D) TPA at 24h.

Figure S5. Viral titers determined by plaque assay after 2 hours of incubation with UiO-66 NPs. ns, *P* *≥* 0.05.

Figure S6. UiO-66 NPs could decrease the expression of DEGs in A549 cells infected with IAV. (A) Venn plots of DEGs from IAV+UiO-66 _vs_ Mock, IAV_vs_Mock and UiO-66 _vs_Mock. (B) Venn plots of DEGs from IAV_vs_UiO-66 , IAV+UiO-66 _vs_IAV and IAV+UiO-66 _vs_UiO-66. (C) Volcano plots of DEGs from IAV_vs_Mock. (D) Volcano plots of DEGs from IAV+UiO-66 _vs_ IAV.


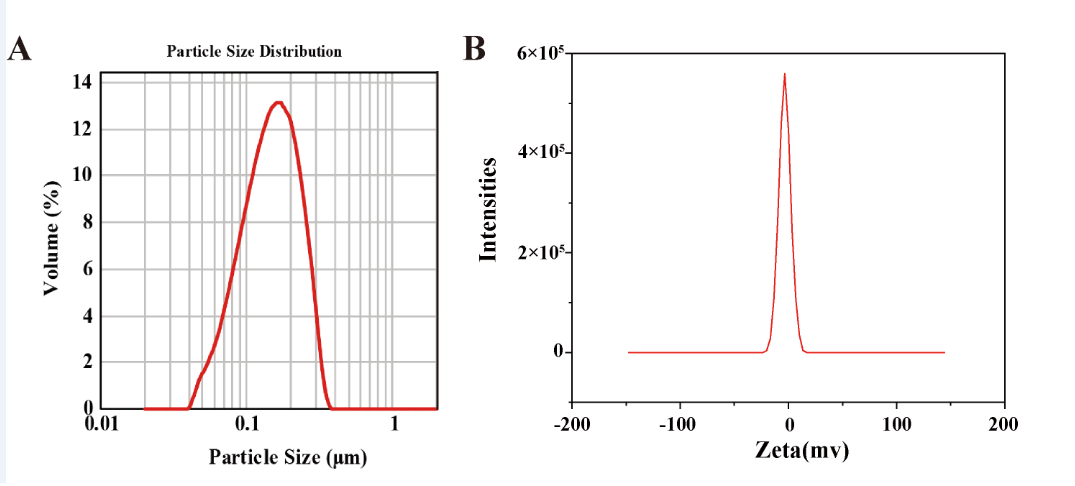

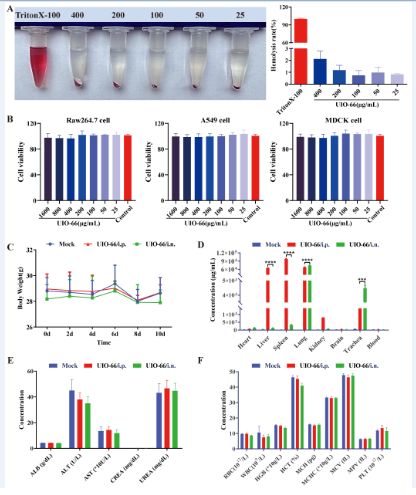


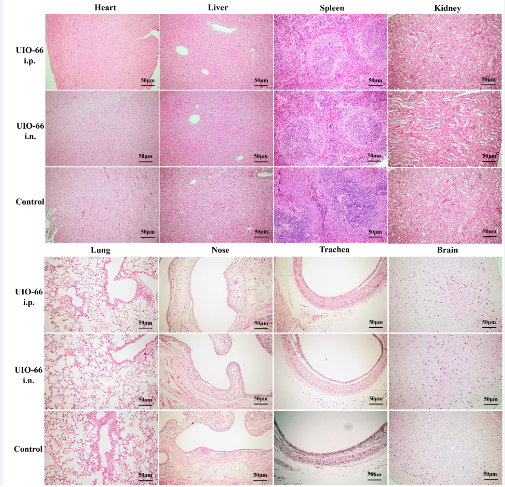


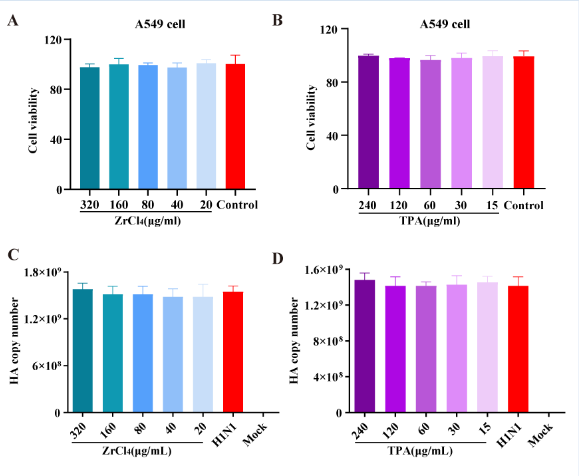


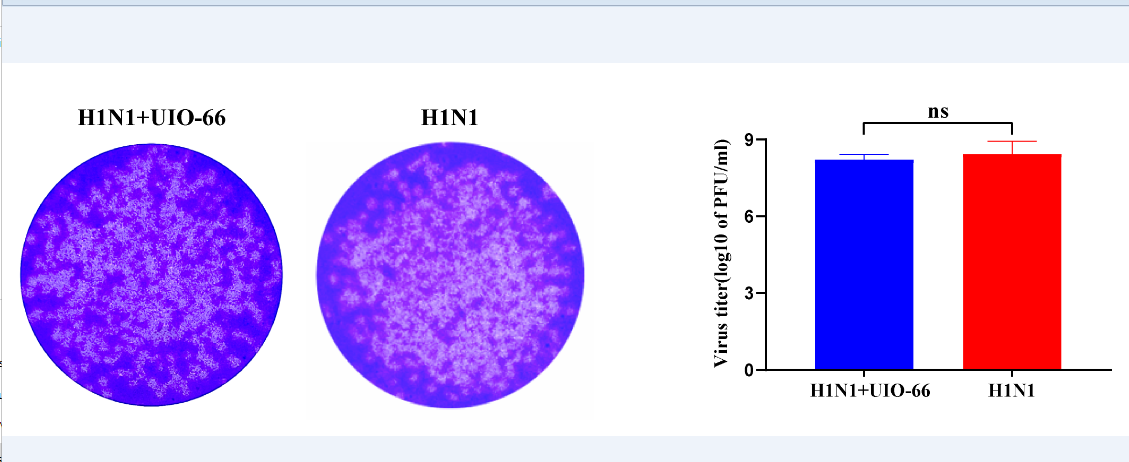


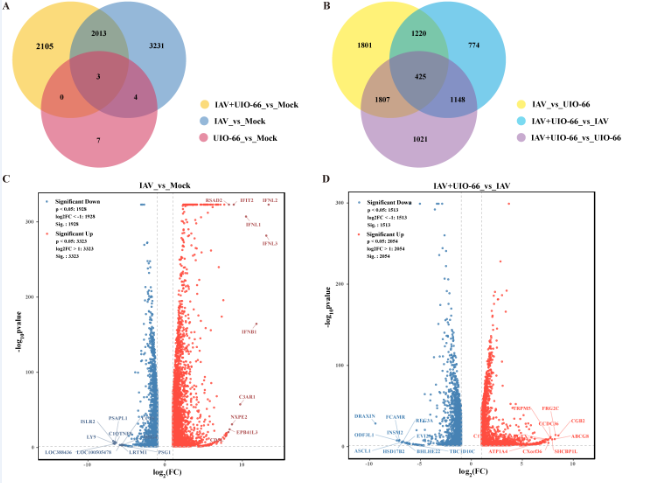

Supplement: Supplementary file 1 — Additional file 1: Table S1. Primers of housekeeping gene β-actin and other targeted genes. Figure S1. A Hydrodynamic size of UiO-66 NPs in water. B Zeta potentials of UiO-66 NPs in water. Figure S2. The evaluation of UiO-66 NPs biocompatibility in vivo and in vitro (UiO-66 NPs, i.n. or i.p.). A Hemolysis rates of UiO-66 NPs. B Cell viability (MTT) of RAW264.7 cells, A549 cells and MDCK cells incubated with UiO-66 NPs for 48 h. C body weight changes of mice (n = 5/group). D Quantitative distribution analysis of Zr4+ in heart, liver, spleen, lung, kidney, brain, trachea and blood samples after treatment with UiO-66 NPs for 14 days in mice. E Serum biochemical indicators of in mice treated with UiO-66 NPs. F Hematological analysis of the mice treated with UiO-66 NPs. Figure S3. Representative hematoxylin and eosin staining of vital organs (heart, liver, spleen, lung, kidney, brain, nose and trachea) at 14 days after treated with UiO-66 NPs. Figure S4. The evaluation of toxicity and effect on HA gene replication of ZrCl4 or TPA in A549 cells. Cell viability (MTT) of A549 cells incubated with A ZrCl4 and B TPA for 48 h at 37 ℃. Analysis of HA replication after IAV infection treated with C ZrCl4 and D TPA at 24 h. Figure S5. Viral titers determined by plaque assay after 2 hours of incubation with UiO-66 NPs. ns, P ≥ 0.05. Figure S6. UiO-66 NPs could decrease the expression of DEGs in A549 cells infected with IAV. A Venn plots of DEGs from IAV+UiO-66 _vs_ Mock, IAV_vs_Mock and UiO-66 _vs_Mock. B Venn plots of DEGs from IAV_vs_UiO-66 , IAV+UiO-66 _vs_IAV and IAV+UiO-66 _vs_UiO-66. C Volcano plots of DEGs from IAV_vs_Mock. D Volcano plots of DEGs from IAV+UiO-66 _vs_ IAV. [file 12951_2024_2358_MOESM1_ESM.docx]
